# Regulatory B Cells: An Exciting Target for Future Therapeutics in Transplantation

**DOI:** 10.3389/fimmu.2014.00011

**Published:** 2014-01-22

**Authors:** Alexandre Nouël, Quentin Simon, Christophe Jamin, Jacques-Olivier Pers, Sophie Hillion

**Affiliations:** ^1^EA2216 “Immunologie, Pathologie et Immunothérapie”, Université de Brest, Université Européenne de Bretagne, SFR ScinBios, LabEx IGO “Immunotherapy, Graft, Oncology”, Brest, France; ^2^Laboratoire d’immunologie, CHRU Morvan, Brest, France

**Keywords:** regulatory B cells, transplantation, immunotherapy, graft, tolerance

## Abstract

Transplantation is the preferred treatment for most end-stage solid organ diseases. Despite potent immunosuppressive agents, chronic rejection remains a real problem in transplantation. For many years, the predominant immunological focus of research into transplant rejection has been T cells. The pillar of immunotherapy in clinical practice is T cell-directed, which efficiently prevents acute T cell-mediated allograft rejection. However, the root of late allograft failure is chronic rejection and the humoral arm of the immune response now emerges as an important factor in transplantation. Thus, the potential effects of Abs and B cell infiltrate on transplants have cast B cells as major actors in late graft rejection. Consequently, a number of recent drugs target either B cells or plasma cells. However, immunotherapies, such as the anti-CD20 B cell-depleting antibody, can generate deleterious effects on the transplant, likely due to the deletion of beneficial population. The positive contribution of regulatory B (Breg) cells or B10 cells has been reported in the case of transplantation, mainly in mice models and highlights the primordial role that some populations of B cells can play in graft tolerance. Yet, this regulatory aspect remains poorly characterized in clinical transplantation. Thus, total B cell depletion treatments should be avoided and novel approaches should be considered that manipulate the different B cell subsets. This article provides an overview of the current knowledge on the link between Breg cells and grafts, and reports a number of data advising Breg cells as a new target for future therapeutic approaches.

## B Cells’ Dual Roles in Transplantation

### Role of B cells in allograft rejection

#### In acute rejection

Key advances in transplant rejection and tolerance came from animal models and proved to be of great importance for subsequent breakthroughs in transplant immunology. In acute rejection, antigen presentation through allorecognition plays a dominant role in initiating the adaptive immune presentation to a CMH-mismatched transplant. Subsequently to allorecognition, a second essential signal is provided by the interaction of costimulatory molecules with their ligands. Thus, B cells can interact with T cells through the CMH-II and B7 family molecules, leading to B and T cell co-activation, differentiation, and antibody (Ab) production. Then, B cells take part into the rejection process by presenting antigens to T cells and by producing donor-specific Abs (1). Costimulatory molecules play a major role in this allorecognition. CD80 and CD86 molecules as well as the inducible costimulatory (ICOS) protein, constitutively expressed on B cells, interact with CD28 on T cells and induce activation and expansion of alloreactive T cells *in vitro* (2). The production of donor-specific alloAbs (DSA) represents another line of evidence of the B cell contribution in acute rejection. Through the generation of opsonized donor cells, B cells enhance T cell alloimmune response and contribute to cellular rejection in a model of skin allograft (3). In this study, the authors demonstrated that polyclonal graft-reactive Abs in the sera of pre-sensitized mice prevented long-term skin graft acceptance in recipients due to the recruitment of complement proteins leading to humoral rejection. Although advances on transplant rejection understanding from animals models are hardly substitutable to humans. Nevertheless B cells have been observed in pediatric biopsy samples (4, 5). These data clearly demonstrated the presence of dense CD20 staining in approximately one third of the 52 biopsy samples from patients with acute rejection and was significantly associated with glucocorticoid resistance and eventual graft failure. In association with molecular analysis of the biopsy profile, it has been demonstrated a strong correlation between CD20^+^ lymphoid aggregates and poor graft outcomes in acute rejection. The presence of B cells infiltrating allografts has been further confirmed in a 4-year follow-up study and found to be associated with reduced graft survival (6). The nature of intragraft B cells has been then explored through immunohistochemical analysis. Cluster-forming CD20^+^ B cells in the rejected grafts are activated and present MHC Class II antigen (HLADR^+^) to CD4^+^ T cells. Some of these clusters contain memory B cells (CD27^+^) (5).

#### In chronic rejection

Acute rejection episodes appear to increase the risks of chronic graft failure development, which is the major complication for long-term allograft survival in humans (7). Indeed, chronic allograft dysfunction in solid transplantation is the principal cause of morbidity and of late allograft loss. A recent evaluation of the short- and long-term renal allograft survival evolution in the United States over 20 years has shown a significant improvement in short-term graft and patient survivals. However, the long-term attrition rates have been slightly improved in spite of arguably more high-risk patients now reaching at least the 1-year mark (8). While, increased immunosuppression has lowered acute rejection rates, it led to more graft loss driven by opportunistic infections or over-immunosuppression (9). Thus, chronic dysfunction remains a universal phenomenon, and not only in the United States (10). Atherosclerosis is defined as a hallmark of chronic allograft dysfunction. The obstruction of the arterial results in ischemia and subsequently in graft loss (11). In an aortic graft mouse model, Wood et al. showed that transplant atherosclerosis does not occur in the absence of the adaptive immune system (12). When alloreactive T cells and B cells are present, transplant vasculopathy is detectable within 30 days of transplantation. Furthermore, local regulation of the destructive immune effectors may be induced by the transfer of *ex vivo* expanded regulatory T (Treg) cells, suggesting that the regulation of the alloimmune response could be impaired in chronic dysfunction (13). Furthermore alloAb production has been demonstrated in human renal transplantation and was found to be predictive of transplant failure (14). Germinal center formation has been described in chronically rejected human heart and kidney, supporting the development of a humoral local immune response (15). In cardiac allograft rejection, infiltration of B cells was also correlated with a higher risk of chronicity of graft failure (16). Altogether, these works clearly suggest that B cells could play pathogenic roles in the course of graft loss.

### Role of B cells in graft tolerance

Considering the overall immunosuppressive function of regulatory B (Breg) cells, particularly in autoimmunity (17), the B cell-mediated alloimmune response could also play a critical role in graft acceptance in transplantation. Experiments with rodent models indicate an accumulation of B cells in tolerant allografts and the formation of germinal centers in transplants with an inhibitory profile (18). In humans, the proportions of whole B cells are increased in transplant-tolerant patients but not in patients under immunosuppressive treatment (19). Furthermore, an increased frequency of the IL-10^+^CD24^+^CD38^high^ immature Breg cells has been observed in tolerant renal-transplanted patients (20). Using microarray approach, the authors demonstrated a molecular signature of tolerance associated with B-cell specific genes. Although this signature may be the consequence of tolerance and not the cause, the authors suggested that B cells could be the primary drivers of the tolerant state. This hypothesis challenges the prevailing paradigm that Treg cells are the principal mediators of transplantation tolerance, and is consistent with emerging data on the role of Breg cells in other immune processes. In line with these observations, large numbers of regulatory CD19^+^CD5^+^CD1d^+^ B cells have been observed in the peripheral blood of patients with long-term kidney graft function (21). In this study, the authors showed that B cells overexpressed the negative B cell receptor FcγRIIb and the negative transcriptional regulator Bank. Moreover, in another group of tolerant patients, peripheral B cells conserved a high capacity to activate the CD40/STAT3 signaling pathway, which promotes high level of IL-10 secretion (22); IL-10 secretion being likely involved in their regulatory activity (23). These results indicate that tolerant patients exhibit B cells with an inhibitory profile, and that some B cell subsets could play a central role in the transplant survey. However, tolerance initiation may be more complicated. Indeed, the depletion of B cells from the PBMC of two patients with high naive B cell counts (one tolerant and one who had received standard immunosuppression) has no effect on the strong, indirect pathway used by T cell regulation (24). The authors propose hypothetical models in which a strictly regulated equilibrium exists between tolerance and rejection. In the conditions of chronic rejection, T cells are less constrained by Treg cells, and cellular immunity is joined by humoral immunity to activate germinal centers’ B cells in order for them to differentiate into DSA-secreting plasma cells. In the state of tolerance, there are critical factors that maintain high levels of regulation in a tissue-specific manner (25). These factors could reside in the increased numbers of circulating B cells, possibly including Breg cells, and the accumulation of Breg cells in the graft.

## Regulatory B Cells in Mice and Humans

### B10 in mouse

#### B10 phenotype

B cells are generally seen as important regulators of the immune responses due to their effector roles giving rise to Ab-producing plasma cells, because they are critical APC that facilitate CD4^+^ T cell activation, and effect multiple other roles in the immune function (26). Nonetheless, the role of B cells in immune systems extends beyond the production of Abs and their Ag-presenting capacities. Over the past decade, novel B cell regulation has been demonstrated in multiple mouse models. The first evidence appeared in 1996 in EAE. Wolf et al. demonstrated that B cells have an incidence in the immune regulation over the course of the disease and contribute to spontaneous recovery (27). B cell-mediated regulation has been further established in intestinal chronic inflammation disease (IBD), collagen-induced arthritis (CIA), and asthma (27–31). Although the identification of different B cell subsets with regulatory functions and the definition of their mechanisms of action are recent events, IL-10-producing Breg cells called B10 cells are the most widely studied Breg cell subset (32).

Numerous studies report contradictory phenotypes of this subpopulation and various B10 profiles have been described in mouse models of autoimmunity. Only a small portion of B cells (1–3% of splenic B cells in wild-type C57Bl6 mice) produced IL-10 after PMA and ionomycin stimulation, implying that not all B cells are able to produce IL-10 (33). The intracellular detection of IL-10 combined with flow cytometric phenotyping have shown that mouse spleen B10 cells are enriched within the small CD1d^high^CD5^+^ B cell subset, where they represent 10–20% of the cells in C57Bl6 mice. This phenotypically unique CD1d^high^CD5^+^ B cell population shares overlapping cell surface markers with a variety of phenotypically defined subsets such as CD5^+^ B1a B cells, CD1d^high^CD23^−^IgM^high^ marginal zone B cells, and CD21^high^CD24^high^ transitional type 2-marginal zone precursor B cells. The production of IL-10 can be attributed to the CD19^+^CD21^high^CD23^high^IgM^high^ transitional B cell subset in the peripheral blood of experimental arthritis (34). The plasticity of IL-10 production makes its usage very difficult as a single marker for B10 cells and depends, in a critical fashion, upon the physiopathological models (resumed in Table 1). Indeed, the functional identification of IL-10-producing B cells may not be restricted to a unique B cell subset and may be a hallmark of an inflammatory micro-environment.

**Table 1:** **Immunophenotype of the regulatory B cells identified in mouse models and human pathologies**.

	Phenotype	Model/pathology	Reference
Mouse	None	EAE	(27)
	None	Chronic colitis	(35)
	CD1d^high^	Chronic colitis	(29)
	CD19^high^	CIA	(36)
	CD21^high^CD23^low^	CHS	(37)
	CD21^high^CD23^high^	CIA	(34)
	CD1d^high^CD5^+^CD19^high^	CHS	(38)
	CD1^hih^CD5^−^CD11b^+^IgM^high^	Chronic colitis	(39)
	Tim-1^+^CD5^+^	Islet allograft	(40)
	CD19^high^CD32b^high^	OVA-TCR Tg	(41)
	CD19^+^CD5^+^CD1d^+^	Pregnancy model of tolerance	(42)
	CX3CR1^+^	Allergy-induced inflammation	(43)
Human	CD24^high^CD38^high^	SLE	(23)
	CD24^high^CD27^+^	Autoimmunity	(44)
	CD5^+^CD24^+^IgD^+^CD38^+^	SLE	(45)
	CD24^high^CD38^high^	ITP	(46)
	CD19^+^CD24^high^CD27^+^	Graves’ disease	(47)
	CD48^+^CD148^+^	Autoimmunity	(48)
	CD19^+^CD38^+^CD147^+^IgM^+^	Cancer	(49)
	CD73^−^CD25^+^CD71^+^	Allergy	(50)

*EAE, experimental autoimmune encephalomyelitis; CIA, collagen-induced arthritis; CHS, contact hypersensitivity; OVA, ovalbumin; TCR, T cell receptor; SLE, systemic lupus erythematosus; ITP, idiopathic thrombocytopenic purpura*.

#### B10 development

The intracellular detection of IL-10 in B cells can be performed both *in vivo* and *in vitro*, in response to various stimuli, which would suggest that IL-10-mediated regulation can be acquired in physiological environments. In an islet allograft rejection model, Breg cells are induced by T cell Ig and mucin domain (TIM) protein-1. They can transfer long-term acceptance in recipients in an IL-10-dependent fashion (40). CD40 engagement appears to be required for the IL-10-dependent Breg cell function in EAE and CIA (29, 36). In transplantation mouse models, allograft tolerance is generated by the administration of anti-CD45RB Ab and is achieved when CD45RB-expressing B cells are present. The induction of tolerance requires that B cell express B7, CD40, and ICAM-1 molecules, suggesting that the B cell-mediated regulatory function is dependent on direct T cell-to-B cell contacts (51). Moreover, several publications emphasize the role of Toll-like receptors (TLR) in B cell-mediated regulation. Mice lacking MyD88, TLR2, or TLR4 exclusively on B cells, develop a chronic EAE (52). CpG (a TLR-9 ligand) can upregulate the production of IL-10 from B cells (53). Thereby, in mice with lupus-like autoimmune disease, splenic B cells with a CD1^high^CD23^−^ marginal zone phenotype can produce IL-10 in response to CpG stimulation (54).

Another key component in the mouse Breg function is the signalings via the BCR. The percentage of Breg was found to have decreased by 90% in MD4 mice with a fixed BCR specific for hen egg lysozyme. Conversely, the overexpression of CD19 and the increased CD40L signaling resulted in an increased number of B10 cells in transgenic mice (55). CD22^-^*^/^*^-^ mice that also ectopically express CD40L show dramatically enhanced numbers of CD1d^high^CD5^+^ B and B10 cells (56). The importance of BCR-related signals is further emphasized by the analysis of the stromal interaction molecules (STIM) 1 and 2. Remarkably, B cells lacking both STIM proteins failed to produce IL-10 after BCR stimulation (31).

More recently, IL-21 has been shown to cause a dramatic increase in the Breg cells frequency and an augmentation of IL-10 secretion (57). These data served to demonstrate that the regulatory B10 cell function requires IL-10 expression, IL-21R signaling as well as CD40, and MHC-II interactions. Finally, it was observed that IL-21 promotes the production of IL-10 and Granzyme B by Breg cells in solid tumor infiltrates (49).

Although IL-10 generally plays immunosuppressive roles during inflammation, IL-10 has also pleiotropic and immunostimulatory activities that opacify the precise role of B10 cells during immune responses. IL-10 represents a key cytokine in Th2-mediated immunity and may be involved in chronic allograft rejection in mice. In a fully mismatched heterotopic mouse heart transplantation model, T-bet and RORγt double-deficient T cells differentiated into alloreactive GATA-3-expressing Th2 cells, which promptly induced allograft rejection characterized by a Th2-type intragraft expression profile (58).

### Human Bregs

All these observations emphasize the importance of B10 cells in mice. The existence of B10 in humans remains unclear and is currently difficult to unify in a coherent model (Table 1). An orthologous IL-10-producing B cell has been described in the CD24^high^CD38^high^ transitional B cell subset (23, 59). Interestingly, this population displays regulatory capacities in healthy volunteers, manifested by the suppression of the Th1 cell differentiation that appears deficient in systemic lupus erythematosus (SLE) patients. Such a defective function of Breg cells could take part into the severity of autoimmune disorders (60). Conversely, SLE B cells spontaneously produced more cytoplasmic IL-10 than control B cells (61). Furthermore, IL-10 can also promote humoral immunity. Human tonsillar B cells co-cultured with CD154-expressing fibroblast secrete large amounts of IL-10, and blockade of the IL-10 function suppress Ig release in a concentration-dependent manner (62). Interestingly, human T cells can activate B cells following CD40L/CD40 interaction and develop their regulatory functions, which are highlighted by an IL-10-independent suppressive effect on T cell proliferation and by the generation of Treg cells (45). More recently, Iwata et al. demonstrated the existence of human IL-10-competent B10 cells noticeable for their ability to secrete IL-10 after *in vitro* stimulation (44). These B10pro B cells were predominantly CD24^high^CD27^+^ and specifically influence the innate monocyte function. Interestingly, both CD24^high^CD27^+^ and CD24^low^CD27^−^ B cells reduced the expression of Th1 cytokine by T cells through an IL-10-independent pathway. Altogether, these evidences demonstrate that human Breg cells may exist beyond IL-10 secretion exclusively. Their precise phenotype and functional capacity need to be further defined and could be critically dependent on the pathology. Furthermore, it could be surmised that B cell-mediated regulation is not restricted to one unique B cell subset but could be triggered by different subset of B cells in association with the physiological contexts.

Several studies have shown that the functional action of Breg cells in human-transplant patients remains ill-defined, and clear identification of the Breg cell subsets is still arduous. A common approach for studying human B cell regulation rely on the analysis of phenotypic transitional B cell subsets in peripheral blood. Although this population is increased in tolerant patients (20, 63) and in kidney transplant recipients with an excellent long-term graft function under immunosuppression (63), no functional data exist that involve Breg populations in the control of the alloimmune response in patients. We recently developed an novel approach to study human B cell regulation. This model led us the possibility to analyze the B cell-mediated suppression of both T cell proliferation and Th1 differentiation in auto- and heterologous cocultures regardless of the phenotype (64). These data demonstrate that Ab-mediated chronic rejection is associated with a defective B cell regulatory function. Furthermore, we posit that, when activated, B cells could have different regulatory capacities based on their maturation status. Discrepancies in the description of homogenous Breg subsets lead us to the conclusion that B cells are able to exhibit functional plasticity that relies on immune regulatory processes to favor either immunity or tolerance.

### B cell-mediated regulation is not exclusively dependent on IL-10

It becomes clear that, in addition to IL-10 production, B cells are able to regulate immune responses through other regulatory processes. Granzyme B, an enzymatic component of the cytotoxic granules, can be produced by B cells. It mediates the cleavage of caspases and initiates the apoptosis of human-infected cells (65). The expression of death-inducing ligands is another B cell-dependent regulatory mechanism. Thus, Fas ligand (FasL) and tumor necrosis factor-related apoptosis-inducing ligand (TRAIL) expressed on mouse and human B cells are able to activate programed cell death in target cells, following ligation with their cell surface receptor (66). Programed death ligand (PD-L) 1 and 2 have also been reported to be expressed by B cells. They can participate in B cell immune suppression. All these data demonstrate that direct cellular contacts without soluble factor are important components for Breg cell activity. Moreover, in mouse EAE model, B cell can regulate CD4^+^CD25^+^ Treg cell expansion via B7 expression, resulting in the suppression of autoimmune inflammation and recovery (67). It seems that human Breg cells have a similar faculty to expand CD4^+^CD25^+^Foxp3^+^ Treg cells and to contribute to the regulation of the Th1 immune response (45). Besides IL-10 secretion, other B cell-secreted soluble factors support this regulatory mechanism. For example, the production of TGFβ can lead to the induction of Foxp3^+^ Treg cells, and thereby, contribute to exert immune suppressive function. *In vitro* experiments using a murine model of allergic airway disease, demonstrated that B cell-secreted TGFβ was involved in the downregulation of lung inflammation and asthma in recipient ovalbumin-sensitized mice after adoptive transfer. Furthermore, TGFβ-expressing B cells induced the conversion of CD4^+^CD25^−^ effector T cells into CD4^+^CD25^+^Foxp3^+^ Treg cells (68). Overall, the immunosuppressive actions of the Breg cells appear complex (69) and cannot be confined only to the secretion of IL-10. Rather, they result from a combined mechanism that associates several cytokines and different cell-to-cell contacts.

Besides, B cells have been shown to secrete natural Abs with immunosuppressive functions in both normal and pathological situations. These Abs can bind to the surface of apoptotic cells. As a result, the phagocytosis efficiency of macrophages and dendritic cells is increased (70). Moreover, these Abs are able to negatively regulate the inflammatory capacity of APC, due to the inhibition of the dendritic cells maturation and the downregulation of molecules involved in antigen presentation (71). The production of Ig appears to be a powerful tool for the control of inflammation. Studies about IVIg clearly demonstrated that a small fraction enriched for a glycoform of IgG possessing sialic acid displayed anti-inflammatory activity in mouse models (72) and in humans (73). The IgG sialylation status may prove to be an important factor in the disease course of alloreactivity. In mouse experiments, T cell-independent immune responses induced suppressive sialylated IgGs, in contrast to T cell-dependent proinflammatory Th1 and Th17 immune responses that induced agalactosylated and asialylated IgGs. Interestingly, the transfer of low amounts of antigen-specific sialylated IgG Abs was sufficient to inhibit B cell activation and pathogenic immune reactions (74). Further studies need to be performed to clarify the nature of alloAbs in models of allograft tolerance and in humoral chronic rejection in humans.

## B Cell-Based Therapeutics

Currently, various drugs targeting T cells are efficiently used to prevent acute rejection in transplantation. However, the efficacy of these treatments is less convincing in chronic dysfunction. A large number of therapeutic options that modify B cell responses are being developed in order to induce tolerance. This useful approach consists in depleting B cells to reduce their possible contribution in alloimmune responses. Numerous cytotoxic Abs binding to B cell surface expressed antigens are available. For example, the anti-CD52 monoclonal Ab Alemtuzumab, is known to prevent acute rejection by depleting T cells and B cells, even though it increases the incidence of humoral rejection in chronic rejection (75). Rituximab (RTX) is a chimeric monoclonal Ab directed toward the pan B-cell surface molecule CD20. It has been shown in some studies that RTX could prevent the emergence of alloAb-producing cells and eliminate short-lived plasma cells; and thereby prove to be clinically effective in the treatment of acute rejection (76).

Nowadays, the involvement of B cells in tolerance might compromise therapeutics targeting B cells Treatment with anti-thymocyte/lymphocyte globulins (ATG) is another approach used to deplete B cells but also T cells and natural killers (NK). ATG induce persistent changes in T cell subsets characterized by low CD4 counts, but they also have deleterious effects on B cell numbers. Interestingly, lymphopenia promotes accelerated atherosclerosis and increases the risk of post-transplantation morbidity (77). The authors suggested that ATG could accelerate immune system aging, characterized by CD4 T cell lymphopenia, CD8 expansion, reduced B cell numbers, and high levels of acute-phase response proteins. This immunosenescence may accelerate rejection. How the decrease of B cells is involved in this process remains to be determined.

Recently, increased levels of B cell activating factor (BAFF) in transplant rejection have been underlined, suggesting a distinct way for therapeutics in transplantation. Indeed, high BAFF-R and BAFF levels are correlated with higher risks of developing graft dysfunction and DSA in stable patients (78) and are associated with increased risks of Ab-mediated rejection (79). Thus, blockade of the BAFF- and APRIL-dependent stimulations of B cells using Atacicept (a recombinant TACI-Ig fusion protein) or Belimumab (a human anti-BAFF monoclonal Ab) could be promising. Indeed, Atacicept can prevent B cell maturation, differentiation, and survival while Belimumab can bind and inhibit soluble BAFF (80).

Despite a growing number of drugs used in transplantation, humoral chronic dysfunction is still a recurrent problem mainly because the precise role of B cells has yet to be completely deciphered. One of the main problems lies in the ability to discriminate regulatory and effector B cells involved in the course of allograft. In keeping with this idea, elimination of B cells with anti-CD20 Ab in mice leads to two contrasting consequences on EAE progression, depending on the time course for B cell depletion. Depletion of B cells before EAE induction exacerbates the severity of the pathology whereas depletion during the acute phase decreases the symptoms (81). Such a striking discrepancy is due to the fact that early B cell depletion before EAE induction eliminates the Breg cell population, and thus, their suppressive role in the disease. In contrast, B cells depletion during the progression of EAE mainly affects effector B cells, which prevents the activation of the CD4^+^ T cells, resulting in the downregulation of the disease severity. This observation is linked to another study in which five patients out of six who had received RTX as induction therapy, developed acute rejection in the first 3 months after transplantation, compared with patients who had received the anti-CD25 Ab Daclizumab (82). Interestingly, patients who received RTX had a higher rate of acute rejection compared to the control group, and also compared to patients who did not received induction therapy. These findings strictly emphasize the importance of B cell-targeting therapeutics and time course of treatments. The balance between effector B cells that promote immunity and Breg cells with potent immunosuppressive role, is a key factor in the microenvironment (83). It becomes evident to considerate not only the time course of the treatments but also to identify the relative contribution of each B cell subset in the evolution of the disease for the development of suitable therapeutic strategies. This is especially important in transplantations where B cell depletion can lead to damaging effects on the transplant (84)

## Perspectives in Transplantation

B cells are crucial regulators of the immunity and their aptitude to generate and maintain tolerance should be exploited for future immunotherapeutic advances in transplantation. Several studies have analyzed the phenotype of circulating T lymphocyte subsets before and after transplantation to evaluate the immunosuppressive effect of conventional treatments. Although the reduction of CD4^+^ and CD8^+^ T cells and the augmentation of CD4^+^CD25^+^Foxp3^+^ Treg cells have been described, there are still conflicting data. Thus, the percentage of CD4^+^CD25^+^CD127^low^ circulatory Treg population is decreasing for years after transplantation despite continuation of the immunosuppressive treatment. In contrast, informations related to B cells are scarce. A decrease of circulating B cell counts after transplantation has been elicited, but the effects of this depletion on graft survival remain unclear (85). However, it is likely that changes in lymphocyte subsets might contribute to the rejection. To this end, analyses of the B cell subsets distribution could be informative to provide an overview of the immune reaction in case of rejection. Recent cellular analyses showed an enrichment of naive and transitional B cells but not of memory B cells in the peripheral blood of tolerant patients without immunosuppressive treatment, compared with immunosuppressed patients with stable graft function (86). We also recently observed decreased frequencies and absolute numbers of activated and transitional B cells but elevated frequencies and absolute numbers of memory B cells in patients with chronic Ab-mediated rejection compared to patients with stable graft function (64). It will be of interest to know which B cell populations are functionally defective in patients with graft rejection and, from a prospective point of view to develop new therapeutics, to have a rigorous follow-up of these populations in transplanted patients before and after grafts.

An alternate method to improve tolerance in transplantation would be to modulate B cell activation. However, the prevention of antigen-specific B cell activation requires the identification of the antigen responsible for BCR stimulation. The blockade of costimulatory receptors like CD19 or CD21, or the stimulation of inhibitory receptors such as CD22 could help achieve beneficial effects through the inhibition of B cell responses. In this context, the humanized anti-CD22 monoclonal Ab, Epratuzumab, has been successfully used in the treatment of SLE. It succeeds in depleting 35% of total B cells while managing to inhibit B cell activation and proliferation (87).

The induction and maintenance of tolerance, based on cellular depletion or on the inhibition of cellular activation, appear to be the most evident B cell-dependent approaches in transplantation. However, it seems relevant now to develop new strategies to trigger the expansion of the Breg population and maintain, or develop, immune tolerance. One possibility could be the *in vivo* generation of Breg cells through the injection of a drug to induce the activation signals for Breg cell differentiation in patients during the transplantation. Yet, more knowledge on the development of Breg cells has to be acquired. An alternative procedure would be the *in vitro* generation of Breg cells, before their transfer into the receiver to elicit efficient regulatory functions. CD40 stimulation could help expand IL-10-secreting positive B cells, which could play an important immunosuppressive role. However, the concomitant activation of effector B cells may oppose the beneficial effects of induced Breg cells. Another possibility to expand Bregs has been recently proposed. The intravenous infusion of apoptotic cells has been shown to prevent arthritis in mice. Activated splenic B cells responded directly to apoptotic cells through the induction of Bregs, which resulted in the generation of IL-10-producing Tregs (30). Consequently, mice were protected from severe joint inflammation and bone destruction. Interestingly, the enhancement of the production of IL-10 was abrogated in the absence of natural IgM (88). Finally, developing therapeutic strategies that exploit Bregs could be key to achieve tolerance in transplantation.

## Conclusion

Diverse mechanisms are involved in graft tolerance but the generation of efficient Breg cells is undoubtedly one of the major aspects. Thus, the rising role of Breg cells, specifically in graft rejection, is now emerging in the current literature. Although our comprehension of animal models is getting more and more accurate, its translation to humans is proving tricky. Many decisive questions on Breg cell biology still need to be addressed. How can their development be controlled? How can their function be induced? Nevertheless, the discovery of Breg cells offers promising and innovative therapeutic approaches, and this area is worthy of pursuit in the near future.

## Conflict of Interest Statement

The authors declare that the research was conducted in the absence of any commercial or financial relationships that could be construed as a potential conflict of interest.
